# Altered Heart Rate Variability in Patients With Schizophrenia During an Autonomic Nervous Test

**DOI:** 10.3389/fpsyt.2021.626991

**Published:** 2021-03-30

**Authors:** Ya Liu, Yuanyuan Huang, Jing Zhou, Guixiang Li, Jun Chen, Zhiming Xiang, Fengchun Wu, Kai Wu

**Affiliations:** ^1^Department of Biomedical Engineering, School of Material Science and Engineering, South China University of Technology, Guangzhou, China; ^2^National Engineering Research Center for Tissue Restoration and Reconstruction, South China University of Technology, Guangzhou, China; ^3^Guangdong Engineering Technology Research Center for Translational Medicine of Mental Disorders, Guangzhou, China; ^4^The Affiliated Brain Hospital of Guangzhou Medical University, Guangzhou Huiai Hospital, Guangzhou, China; ^5^Department of Scientific Research, Institute of Health Medicine, Guangdong Academy of Sciences, Guangzhou, China; ^6^Guangdong Engineering Technology Research Center for Diagnosis and Rehabilitation of Dementia, Guangzhou, China; ^7^Department of Radiology, Panyu Central Hospital of Guangzhou, Guangzhou, China; ^8^Key Laboratory of Biomedical Engineering of Guangdong Province, South China University of Technology, Guangzhou, China; ^9^Department of Nuclear Medicine and Radiology, Institute of Development, Aging and Cancer, Tohoku University, Sendai, Japan

**Keywords:** autonomic nervous system, nonlinear dynamics, stress, recovery, heart rate variability, schizophrenia

## Abstract

Reduced heart rate variability (HRV) and dysfunction of the autonomic nervous system (ANS) have been observed in schizophrenia patients. HRV parameters of schizophrenia patients in the resting state have been well-documented; however, these parameters of schizophrenia patients who experience continuous psychophysiological stress remain unclear. The objective of this study was to systematically explore the linear and nonlinear HRV parameters between schizophrenia patients and normal controls and to detect the adaptive capabilities of HRV of schizophrenia patients during the stimulation tests of autonomic nervous system. Forty-five schizophrenia patients and forty-five normal controls, matched for age, sex and body mass index, completed a 14 min ANS test. Thirteen linear and nonlinear HRV parameters of all subjects under the ANS test were computed and statistically analyzed between groups and between sessions. The STROBE checklist was adhered to in this study. All time-domain HRV features in the ANS test were significantly different between schizophrenia patients and normal controls (*p* < 0.01). The schizophrenia patients showed significantly low values in the Poincaré indices, which revealed significantly decreased heart rate fluctuation complexity compared with that of normal controls (*p* < 0.001). In addition, the normal controls, not schizophrenia patients, showed significant differences between the recovery and stress states in the parameters of low frequency, high frequency, and nonlinear dynamics. Schizophrenia patients showed autonomic dysfunction of the heart in a series of stimulation tests of the autonomic nervous system and could not regain normal physiological functions after stress cessation. Our findings revealed that the dynamic parameters of HRV in psychophysiological stress are sensitive and practical for a diagnosis of schizophrenia.

## Introduction

Decreased variability in the heart rate of patients with schizophrenia in the resting state has been reported in previous studies (1–4). Reduced heart rate variability (HRV) has been shown to be related to a series of risk factors for cardiovascular disease and psychophysiological stress (5, 6). Stress is a kind of physiological response to threatening environments and results in negative emotion and an increased heart rate (6). It appears to play a major role in the pathophysiology of nearly all psychiatric disorders (7). Patients with schizophrenia experiencing more negative emotions in their daily lives (8). In psychotic patients with a predominance of negative symptoms, both the cognitive and emotional components of executive functions are affected (9). The autonomic nervous system (ANS) is one of the key systems involved in the generation of these physiological changes (10). The regulation of the central nervous system in cognitive function influences peripheral ANS activity (11). The cardiac autonomic dysregulation of schizophrenia patients results in emotional and cognitive dysfunctions (12) and decreased HRV (13–15).

Previous studies have indicated that the measurements of some psychological features can reflect the flexible and adaptive capacity of autonomic function when an individual faces environmental change and psychological stress (16). HRV, as a beat-to-beat variation in heart rate, is regulated by sympathetic and parasympathetic systems. In addition, it can be influenced by worry, relaxation, and other variables such as thermal, stress task-induced, and so on (17–20). It can serve as an index to level the balance of ANS activity, and the parameters of HRV seem to be more sensitive in autonomic reactivity stress (21, 22). Low cardiac vagal tone is an index of impaired central-peripheral neural feedback mechanisms (23). Internalizing psychopathology and dysregulated negative affect are characterized by dysregulation in the autonomic nervous system and reduced heart rate variability (HRV) because of increases in sympathetic activity alongside reduced vagal tone (2, 24). Psychological stress-related measures of HRV have been studied for assessing autonomic function of individuals with mental and physical health problems (16). Castro (25) demonstrated that spectral and non-spectral HRV analyses of schizophrenia patients have similar responses to mental stress, resulting in autonomic changes in sympathetic activation and parasympathetic inhibition.

Notably, most of the abovementioned studies focus on HRV features of schizophrenia patients in the resting state. However, the linear and nonlinear HRV parameters of schizophrenia patients are not systematically studied in a stress test and a recovery period. The RR time series data from the sinus RR intervals are a physiological system, and their nonlinear characteristics may indicate greater complexity and flexibility than linear characteristics (26). In this study, we aimed to compare the linear and nonlinear HRV parameters of schizophrenia patients with those of normal controls via the ANS test, including resting, mental arithmetic, deep breathing, and recovery states. The function of the cardiac autonomic system was estimated by analyzing the stress test and recovery state, and the application of HRV measurement as a quantitative marker of cardiac autonomic regulation in the physiological and psychological environment of schizophrenia patients was explored.

## Materials and Methods

### Participants

Ninety Chinese participants aged 18–65 years old were included in this study. The study was carried out in compliance with the Declaration of Helsinki and approved by the ethics committee of the Affiliated Brain Hospital of Guangzhou Medical University. And all participants signed informed consent. The control group and patients consisted of forty-five participants each. Patients with schizophrenia were recruited from Guangzhou Brain Hospital, China and diagnosed by psychiatrists based on diagnostic criteria (Diagnostic and Statistical Manual of Mental Disorders- IV-Text Revision, DSM-IV-TR) (27). Demographic information, including age, age of onset, disease duration and marital status, and their duration of illness is recorded in Table 1.

**Table 1:** Participant characteristics.

	**Normal controls (mean ± SE)**	**Schizophrenia patients (mean ± SE)**	***p*-value**
*N*	45	45	/
Sex (female/male)	(22/23)	(17/28)	0.626
Age (years) ± SD	38.8 ± 15.3	42.9 ± 13.0	0.193
BMI(kg/m^2^)	22.4 ± 3.2	24.6 ± 3.9	0.004
Education in years	14.0	11.0	0.001
Handedness	Right	Right	/
duration of disease (years)		15.5 ± 11.1	/

*SE, standard error; SD, standard deviation; BMI, body mass index*.

To determine eligibility, all normal controls conducted routine clinical tests in school infirmary and psychiatric diagnostic cognitive testing before taking the ANS test. Forty-five normal controls from the university and community were recruited as the control group and matched to patients by age, sex and handedness. The demographic information, including age, age of onset, disease duration and marital status, was recorded in Table 1. All participants were right-handed. None of them had other illnesses affecting the ANS, such as heart disease, high blood pressure, diabetes or other mental disorders. All of them did not meet DSM-IV-TR diagnosis of any mental illness, and have no family history of mental illness as well use any medication. Pregnant females, females during follicular and luteal phase of the menstrual cycle and people who had substance abuse were also excluded. All participants were instructed to refrain from caffeine, alcohol or high strength exercise training on the day before the study.

### Study Design

To assess autonomic function, resting, stressed and recovered heart rates related to parasympathetic and sympathetic stimulations were obtained in the study (28). The ANS test consisted of four steps. (1) In the resting state, participants remained seated and kept spontaneously breathing for 4 min. (2) In the mental arithmetic state, participants estimated parity and counted the odd number or even number mentally when the computer played serially every 2 s. Then, the participants told the odd number or even number when the playing ended. This mental stress lasted for 3 min. (3) In the deep breathing state, subjects inhaled for 5 s and then exhaled for another 5 s. The deep breathing was repeated six times per minute and the test lasted 3 min. (4) In the recovery state, following the collection of stress ECG recordings, participants remained seated and kept spontaneously breathing for a 4-min recovery period. Every participant would be allowed to relax for 30 s between each state. The ANS test continued for 14 min and concluded with audio prompts. Stress and anxiety were not evaluated during the ANS test.

### Data Acquisition

The signal data were obtained during working time (9:00–12:00 a.m. and 2:00–6:00 p.m.), for the HRV parameters we used has shown no significant differences between day and night (29). All participants were instructed to keep seated in a quiet room during the 14-min period of continuous ECG data recording which would be used in statistical analysis. Every participant was instructed to relax for more than 10 min when seated in the chair. Prior to the beginning of the experiment, the audio played the test's general procedures and announcements.

Electrocardiography (ECG) signals were recorded by using a two-lead ECG device (SYMTOP INSTRUMENT CO., LTD, Beijing, China) with a sampling rate of 1,000 Hz. HRV was derived by extracting the peaks of the R waves of the ECG signals and measuring the beat-to-beat intervals of the heart. Continuous RR intervals were calculated by the Pan Tompkins algorithm on the basis of digital analyses of the slope, amplitude, and width of the RR waves (30). Errors in R-peak location and RR intervals were carefully corrected by manual checks (31). The HRV parameters were calculated with KUBIOS HRV software, version 2.2 (released by the University of Eastern Finland). HRV analyses were divided into different segments of RR interval data under four test experimental conditions.

Three types of measurements for HRV signals were analyzed to evaluate ANS function, including time domain, frequency domain and nonlinear dynamics. The time domain features include the mean values of each RR interval (MEAN), the standard deviation of normal-to-normal intervals (SDNN), the root mean square successive differences (RMSSD) that reflect short-term oscillations of HRV, and the percentage of normal-to-normal intervals more than 50 ms (pNN50). The frequency power was obtained after cubic spline interpolation and Fourier transformation of HRV signals. The features of frequency domain are divided into four parts: VLF power (0.003–0.04 Hz), LF power (0.04–0.15 Hz), HF power (0.15–0.4 Hz) and LF/HF ratio. The SDNN and LF reflect sympathetic and parasympathetic activities. RMSSD and HF power are viewed as traditional parasympathetic markers and reflect parasympathetic cardiac controls (31).

Nonlinear phenomena of HRV signals are involved in complex interactions of autonomic and central nervous regulations (31). The most commonly used nonlinear parameters of HRV include the Poincaré plot (SD1 and SD2), detrended fluctuation analysis (DFA, a1 and a2), sample entropy (SampEn), approximate entropy (ApEn) and the correlation dimension. In the Poincaré plot, the graph represents the correlation of successive heartbeat intervals. There are two parts to the Poincaré plot: SD1 describing short-term variability and SD2 describing long-term variability of the RR intervals (32). SD1 is highly correlated with the RMSSD of time domain (33). The visible changes of SD1 and SD2 in the shapes of Poincaré plot describe variable RR intervals of the cardiac time series (34), and SD1 is regarded as a marker of parasympathetic functioning (34, 35). In DFA, distinct range scaling exponents of beat-to-beat fluctuations account for regulating the mechanism of cardiac dynamics (36). DFA uses scaling coefficients (α) to measure the correlation of heart-beats in a non-stationary temporal sequence. The most commonly used α ranges are short-range scaling (α1) and long-range scaling (α2) (37). α1 reflects sympathetic activity, while α2 is related to sympathetic and parasympathetic activities (38). In previous studies, α1 and α2 were utilized to assess autonomic nervous system activity and investigate interactions with the hypothalamic-pituitary-adrenal axis (39). The model of ApEn represents the regularity and complexity changes of the time series; if the patterns in a successive series are close, regularity remains high, and complexity is small, corresponding to lower ApEn values (40). ApEn is a quantified measurement of parasympathetic modulation. Karl-Jürgen Bär found that the ApEn of RR intervals for SZ patients is significantly lower than that of controls, which indicates that the complexity of heart rate of schizophrenia patients is decreased. We used ApEn to measure the nonlinear complexity of HRV in acute schizophrenia, and the logistic regression model showed that ApEn was a significant predictor for diagnosing schizophrenia to some extent (4).

In summary, sixteen kinds of features were calculated, including MEAN, SDNN, RMSSD, PNN50, VLF, LF, HF, LH/HF, SD1, SD2, a1, a2, and ApEn.

### Statistical Analysis

All statistical tests were accomplished by using IBM SPSS Statistics version 25. The statistical analysis included three steps. First, sex distributions between schizophrenia patients and normal controls were matched with a Pearson Chi-squared test. Years of education were tested with an independent-samples *t*-test. Second, time domain, frequency domain and nonlinear dynamics parameters of HRV of the two groups in the four states were calculated. Differences in each HRV parameter between patients and the control group were tested with a two-tailed paired Student's *t*-test when the data normally distributed. When the data were not normally distributed, we used the nonparametric test (Mann-Whitney U-test). The relationship between resting HRV and age was further tested and validated using Pearson rank correlations. Third, the variations between groups and that between states in each group (within-subjects) were analyzed by using ANOVA to comparing each HRV parameter from autonomic test sessions, respectively, and Welch analysis was applied when equal variance was not assumed. Moreover, the Holm-Bonferroni correction was applied to the HRV parameter between groups. Significance for all statistics was defined as *p* < 0.05.

## Result

### Initiative Participant Characteristics

All demographic characteristics of the participants and the clinical characteristics of the patients are recorded in Table 1. There were no significant differences in age and sex between patients and the control group. Groups differed in years of education achieved and body mass index (BMI); schizophrenia patients had greater BMI values than controls.

### Comparisons of HRV Features Between Groups

In this study, HRV signals of ninety participants under the ANS test were collected. Time and frequency domains and nonlinear characteristics of HRV signals were calculated. Fifty-two HRV features are shown in Table 2, with thirteen individual features for each testing state. As statistic 1 showed that most HRV parameters of schizophrenia patients were reduced during the stimulation tests. The reduction represented an overall decrease in autonomic nervous system activity. By comparing the HRV features of the schizophrenia patients with those of normal controls, the results revealed that some parameters differed significantly. In the resting state, all time-domain features (*p* < 0.001) and VLF, HF, and Poincaré (*p* = 0.001) of the schizophrenia patients were much lower than those of normal controls (the effect size of them almost > 0.5, except that of VLF, LF/HF ratio and α1). The LF/HF ratio of the schizophrenia patients was significantly higher than that of normal controls. In the mental arithmetic state, the MEAN, SDNN, RMSSD, pNN50, and Poincaré (*p* < 0.001) of the schizophrenia patients were significantly lower than those of normal controls (the effect size of them almost > 0.5, except that of MEAN). In deep breathing, all time-domain features (*p* < 0.001) and VLF, LF, HF, and Poincaré (*p* < 0.001) of the schizophrenia patients were much lower than those of normal controls (the effect size of them almost > 0.5, except that of VLF). In the recovery state, the MEAN, SDNN, RMSSD, pNN50, and Poincaré (*p* < 0.001) of the schizophrenia patients were significantly lower than those of normal controls (the effect size of them all > 0.5).

**Table 2:** Sample characteristics and analyses of between-group differences.

		**Resting**				**Math arithmetic**			
**Features**	**Unites**	**Normal control**	**Schizophrenia patients**	***p***	**Cohen's *d***	**Normal control**	**Schizophrenia patients**	***p***	**Cohen's *d***
MEAN	ms	841.81 ± 128.86	738.48 ± 109.63	0.000	0.863	770.73 ± 141.68	711.98 ± 102.38	0.027	0.475
SDNN	ms	40.24 ± 19.08	23.20 ± 12.88	0.000	1.047	46.90 ± 21.69	24.55 ± 15.40	0.000	1.188
RMSSD	ms	37.42 ± 21.97	17.26 ± 13.37	0.000	1.109	31.71 ± 18.21	16.92 ± 13.13	0.000	0.609
pNN50	%	19.79 ± 20.06	3.54 ± 9.99	0.000	1.025	13.29 ± 15.70	3.26 ± 8.80	0.000	0.788
VLF	ms^2^	416.72 ± 439.77	289.31 ± 367.47	0.000	0.314	784.07 ± 928.06	309.27 ± 794.09	0.414	0.550
LF	ms^2^	407.72 ± 644.70	157.43 ± 191.43	0.926	0.526	301.53 ± 354.25	141.59 ± 285.96	0.592	0.497
HF	ms^2^	826.15 ± 1,038.37	164.87 ± 268.06	0.000	0.872	617.28 ± 945.56	155.35 ± 254.48	0.201	0.667
LF/HF	N/A	1.06 ± 1.63	2.05 ± 3.07	0.002	0.403	1.09 ± 1.67	2.27 ± 4.84	0.089	0.326
α1	/	0.88 ± 0.30	1 ± 0.33	0.071	0.381	0.95 ± 0.30	1 ± 0.31	0.465	0.164
α2	/	0.82 ± 0.30	1.01 ± 0.28	0.002	0.655	0.94 ± 0.26	0.96 ± 0.31	0.742	0.102
SD1	ms	26.57 ± 15.60	12.24 ± 9.51	0.000	1.109	22.53 ± 12.94	12.01 ± 9.33	0.000	0.933
SD2	ms	49.60 ± 23.45	29.96 ± 16.41	0.000	0.970	61.51 ± 29.18	32.11 ± 20.30	0.000	1.170
ApEn	/	0.77 ± 0.21	0.91 ± 0.23	0.002	0.636	0.72 ± 0.11	0.74 ± 0.12	0.270	0.174
		**Deep breath**				**Recovery**			
**Features**	**Unites**	**Normal control**	**Schizophrenia patients**	***p***	**Cohen's** ***d***	**Normal control**	**Schizophrenia patients**	***p***	**Cohen's** ***d***
MEAN	ms	822.97 ± 131.17	724.63 ± 110	0.000	0.8124	812.21 ± 142.41	725.89 ± 102.72	0.003	0.695
SDNN	ms	64.96 ± 28.43	31.16 ± 20.77	0.000	1.358	36.06 ± 14.34	23.42 ± 12.14	0.000	0.951
RMSSD	ms	50.68 ± 27.53	21.99 ± 17.70	0.000	1.240	23.50 ± 14	14 ± 9.21	0.003	0.802
pNN50	%	24.76 ± 17.39	5.97 ± 11.25	0.000	1.283	6.30 ± 9.78	2.16 ± 5.90	0.000	0.513
VLF	ms^2^	540.46 ± 577.83	289.18 ± 427.39	0.001	0.494	439.50 ± 341.02	244.22 ± 305.18	0.373	0.603
LF	ms^2^	2,485.64 ± 2,156.66	552.39 ± 930	0.005	1.164	515.08 ± 546.14	214.73 ± 350.18	0.118	0.655
HF	ms^2^	1,664.59 ± 1,535.76	299.91 ± 440.52	0.01	1.208	300.97 ± 582.08	102.06 ± 182.41	0.831	0.461
LF/HF	N/A	1.70 ± 1.09	2.03 ± 2.13	0.894	0.195	4.03 ± 4.28	4.66 ± 6.12	0.732	0.119
α1	/	1.16 ± 0.26	1.16 ± 0.36	0.850	0	1.21 ± 0.31	1.12 ± 0.40	0.207	0.252
α2	/	0.68 ± 0.29	0.87 ± 0.35	0.007	0.591	0.97 ± 0.40	1.07 ± 0.41	0.239	0.247
SD1	ms	36.02 ± 19.58	15.62 ± 12.58	0.000	1.240	16.71 ± 9.96	9.94 ± 6.55	0.000	0.803
SD2	ms	84.10 ± 36.49	40.84 ± 27.20	0.000	1.344	47.68 ± 18.85	31.13 ± 16.62	0.000	0.931
ApEn	/	0.61 ± 0.10	0.69 ± 0.12	0.000	0.724	0.69 ± 0.13	0.68 ± 0.09	0.859	0.089

*MEAN, Mean value of R-R wave interval; SDNN, Standard deviation of normal-to-normal interval; RMSSD, Root mean square successive difference; PNN50, Percentage of normal-to-normal interval more than 50 ms; VLF, very Low frequency power; LF, Low frequency power; HF, High frequency power; LF/HF, The ratio of low frequency power and high frequency power; SD1, The long axis of ellipse regarding RR interval; SD2, The short axis of ellipse regarding RR interval; α1, Short-term fluctuation slope; α2, Long-term fluctuation slope; ApEn, The approximate entropy*.

*The results of person correlation analysis showed that age was not significantly associated with the RR time series in normal controls and schizophrenia patients (p > 0.1)*.

These results indicated that most HRV parameters of schizophrenia patients were reduced compared with those of normal controls in the ANS test.

### Comparisons of HRV Features Between States

As statistic II showed, the variations of four sessions in the ANS test were individually analyzed. Some features of HRV signals in two groups changed significantly in different states (*p* < 0.05).

**Figure 1:**
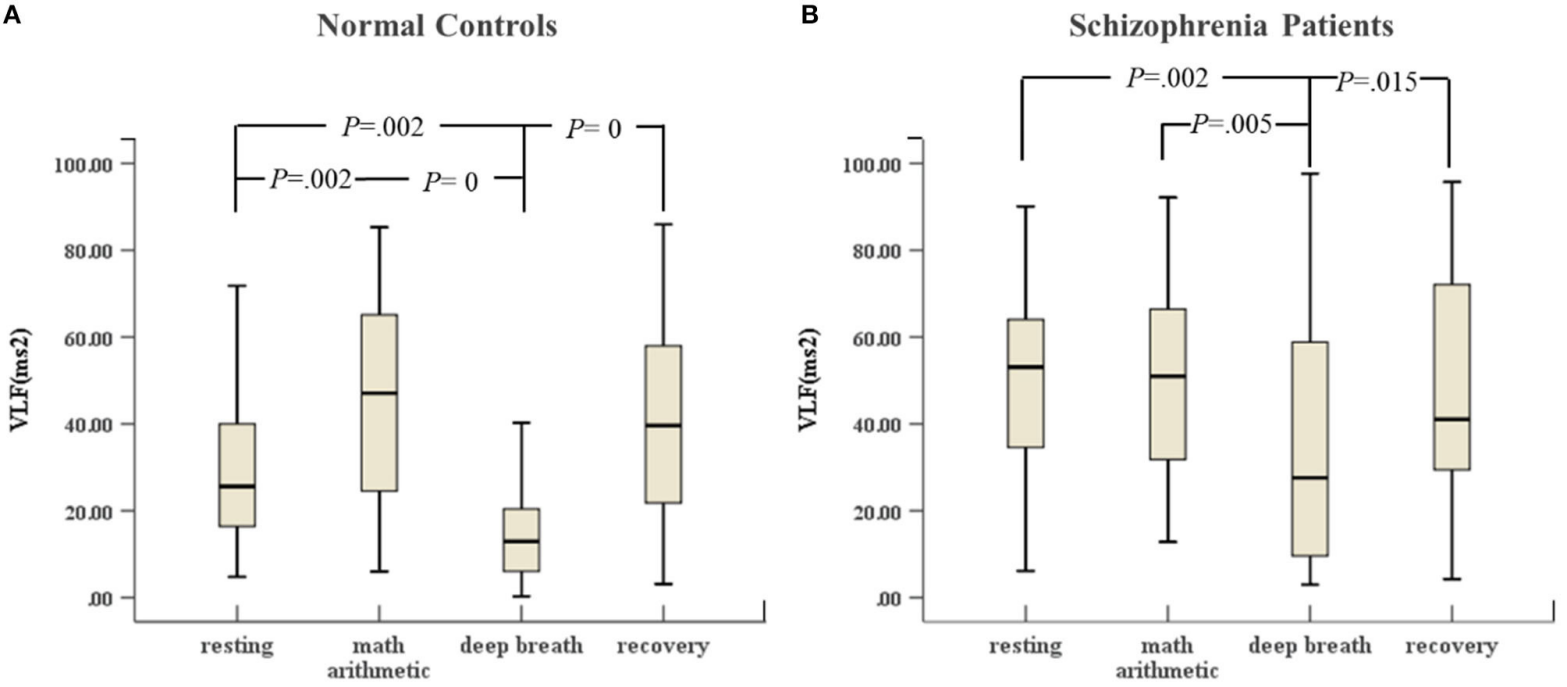
**(A)** VLF of HRV of normal controls (*n* = 45) and **(B)** VLF of HRV of schizophrenia patients (*n* = 45) during the ANS test, which was analyzed with ANOVA, *p* < 0.05.

As shown in Figure 1, schizophrenia patients and normal controls behaved similarly in the ANS test. The VLF in the deep breathing state was significantly lower than that in the resting state, mental arithmetic, and recovery states. Figure 1A shows that the variance of VLF of normal controls from the resting state to mental arithmetic state increased noticeably, but Figure 1B reveals that the VLF of the schizophrenia patients in the mental arithmetic state was similar to that of controls.

**Figure 2:**
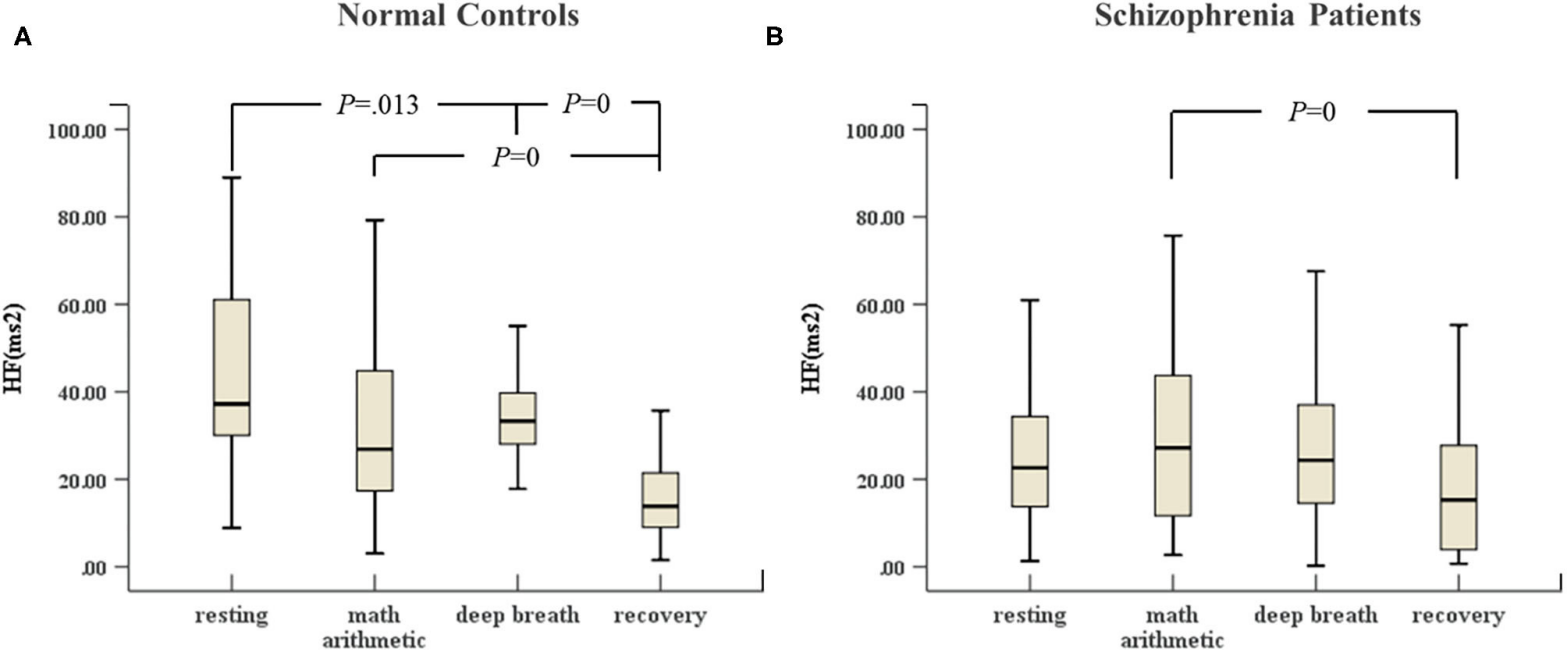
**(A)** HF of HRV of normal controls (*n* = 45) and **(B)** HF of HRV of schizophrenia patients (*n* = 45) during the ANS test, which was analyzed with ANOVA, *p* < 0.05.

In Figure 2, the HF of normal controls in deep breathing state changed significantly compared to resting state and recovery state, and the HF increased in the deep breathing state, but Figure 2B reveals that the HF of the schizophrenia patients in the deep breathing and recovery states did not change significantly.

**Figure 3:**
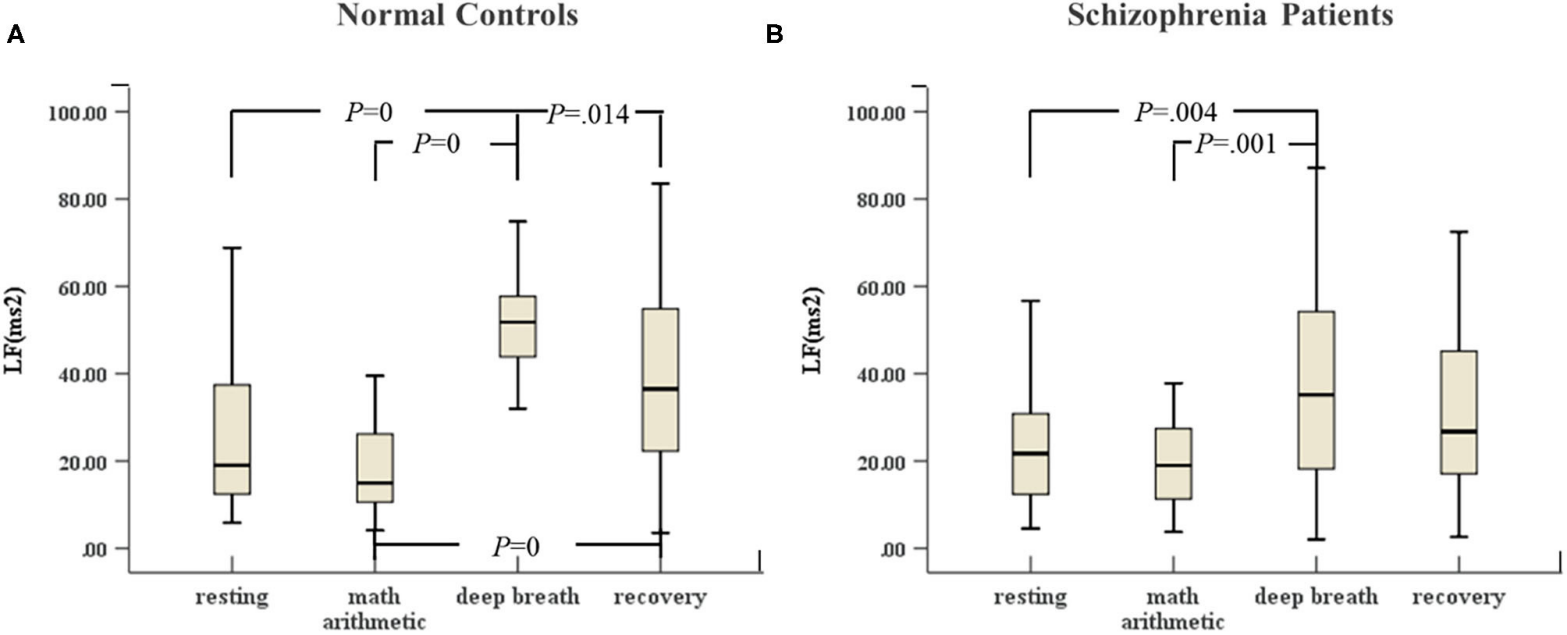
**(A)** LF of HRV of normal controls (*n* = 45) and **(B)** LF of HRV of schizophrenia patients (*n* = 45) during the ANS test, which was analyzed with ANOVA, *p* < 0.05.

**Figure 4:**
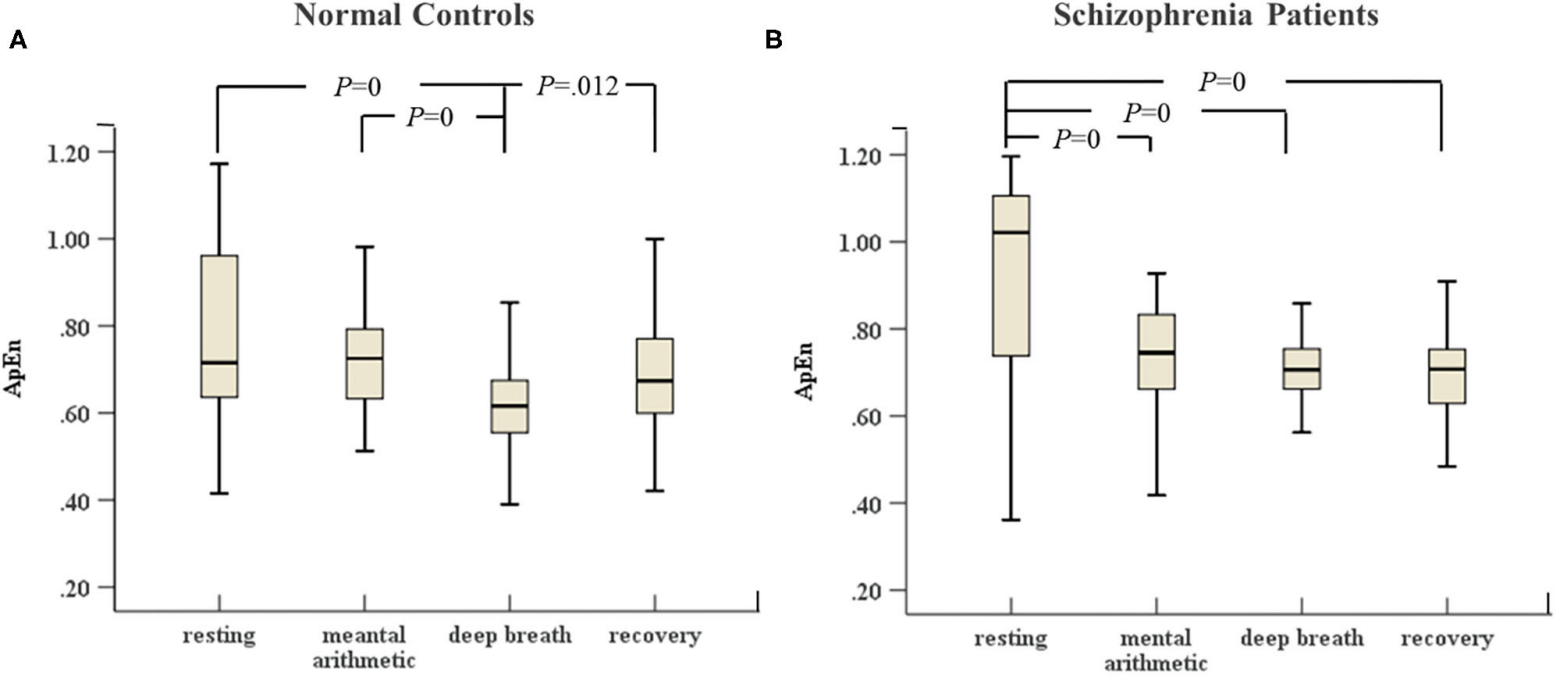
**(A)** ApEn of HRV of normal controls (*n* = 45) and **(B)** ApEn of HRV of schizophrenia patients (*n* = 45) during the ANS test, which was analyzed with ANOVA, *p* < 0.05.

In Figures 3, 4, the variances of LF and ApEn of normal controls under the ANS test were different compared to the variances of these parameters in schizophrenia patients, especially in the recovery state. Figures 3A, 4A show that the values of LF and ApEn of normal controls in the recovery state were significantly different from those in deep breathing state. However, Figures 3B, 4B demonstrate that the LF and ApEn of schizophrenia patients between deep breathing state and recovery state were not significantly different. In contrast to normal controls, schizophrenia patients showed prolonged stress activation in Figure 4B, which displayed sustained decreases in ApEn and decreased ApEn throughout the recovery state.

**Figure 5:**
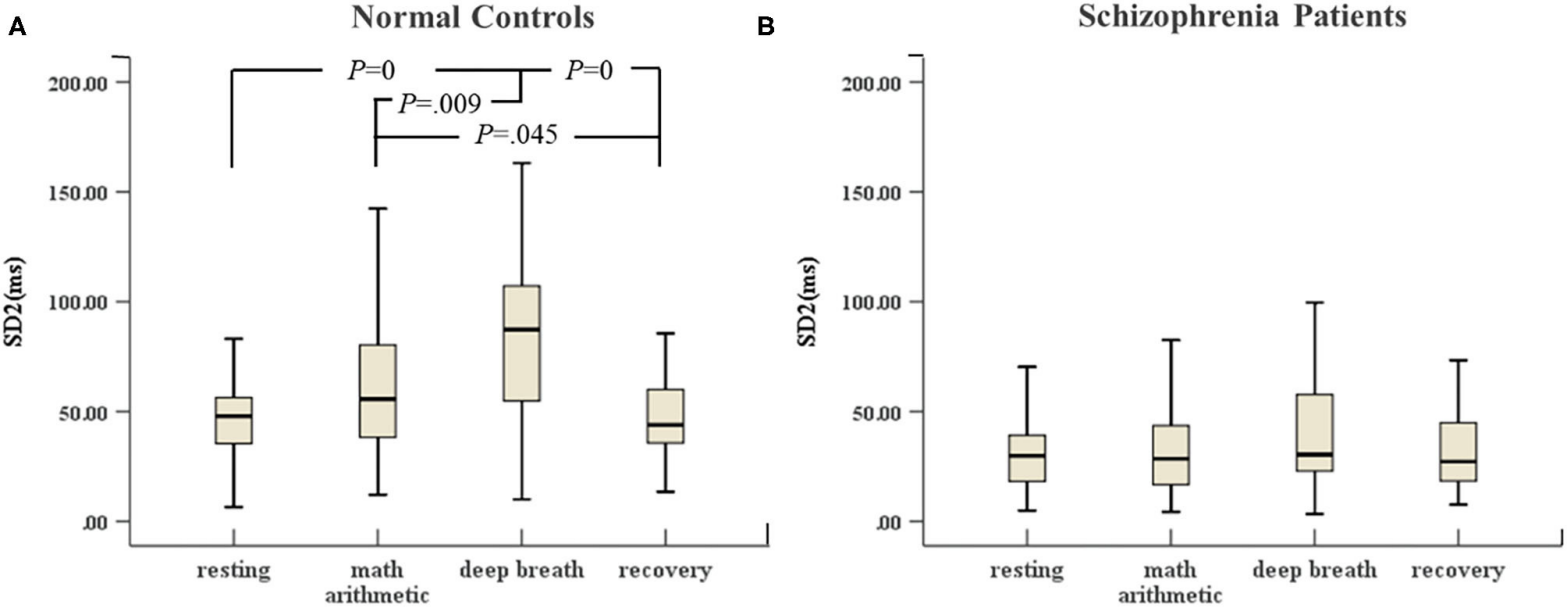
**(A)** SD2 of HRV of normal controls (*n* = 45) and **(B)** SD2 of HRV of schizophrenia patients (*n* = 45) during the ANS test, which was analyzed with ANOVA, *p* < 0.05.

In Figure 5A, there were great differences between the stress period and resting state (including recovery) in the control group. SD2 in the deep breath state was highest compared with the other three stimulation tests. SD2 in mental arithmetic was significantly different compared with that in the recovery state. However, Figure 5B shows that SD2 of the schizophrenia patients did not change significantly in the four sessions of ANS test.

## Discussion

In this study, we explored the difference in HRV parameters between schizophrenia patients and normal controls and, for the first time, assessed the autonomic nervous system of schizophrenia patients from the stress test to recovery by using linear and nonlinear dynamic analysis methods for HRV. The results indicated that schizophrenia patients did not recover from the stress activation state after stress cessation. The results also demonstrated that the dynamic methods for HRV analysis exhibited similar sensitivity compared to linear methods for individuals in response to the stimulation tests of the autonomic nervous system.

Julian F. illustrated the relationship between HRV and regional cerebral blood flow and highlighted that HRV may serve as an important organism function index associated with adaptability and health (16). Low HRV is found in poor attention regulation, physiological flexibility and affective information processing. Thus, individuals are not able to appropriately adjust their responses to changing task demands when vagal-mediated HRV is decreased (23). The cognitive dysfunction of schizophrenia patients is related to the severity of psychopathology in schizophrenia (12). In particular, the severity of specific symptoms of patients in schizophrenia is significantly relevant to reduced HRV features (41).

The main findings between schizophrenia patients and normal controls under the ANS test in the study are as follows. (1) Between-groups: In all four testing states, most HRV features of schizophrenia patients were lower than those of normal controls, especially time domain features and Poincaré, which exhibited highly significant differences. (2) Within-subjects: (a) No significant differences were found between resting state and mental arithmetic state, except for the VLF, which demonstrated a significant increase in only normal controls, not in schizophrenia patients; (b) The LF, HF, ApEn and SD2 of normal controls showed significant differences between resting, deep breathing and recovery states. However, in schizophrenia patients, we did not observe the recovery of autonomic system after stress termination. The specific discussions are further elaborated below.

Studies of HRV in the resting phase have identified decreased levels of HRV in patients with schizophrenia (1, 42, 43). In the resting state, the RMSSD and HF of schizophrenia patients are significantly lower than those of normal controls, reflecting decreased parasympathetic activity (1, 22, 44–46). Several studies have suggested a pathophysiological link between central autonomic dysfunction and symptoms of schizophrenia and that these could be heritable. There are similar alterations during the arithmetic stress task. To stress termination, it was found that prolonged reaction to mental arithmetic stress in the first-degree relatives of schizophrenia patients (47). It is consistent with our findings. The balance of autonomic system tends to shift toward the activation of the sympathetic system and the withdrawal of the parasympathetic system in the mental arithmetic state. In the deep breathing state, the parasympathetic nervous system is stimulated and thus leads to increased parasympathetic activity. We increased the breathing load to enhance parasympathetic activity in this study. Our results showed that the RMSSD, HF and SD1 of schizophrenia patients, which reflect parasympathetic activity (34, 35), were significantly lower than those of normal controls in the ANS test. This confirmed that the parasympathetic system of patients did not activate normally, as it did for normal controls, when the stimulation was conducted. Meanwhile, the LF and SD2 of schizophrenia patients, which are commonly modulated by changes in the sympathetic and parasympathetic tone, were significantly lower than those of normal controls. A higher LF/HF ratio of schizophrenia patients was also found in this study. The increased HRV-LF/HF indicates the higher sympathetic nervous activity in comparison to parasympathetic nervous activity, which may result in a less flexible autonomic system (48). This observation suggested that the dysfunction of autonomic nervous system of schizophrenia patients occurred. With these results, schizophrenia might be related to decreased vagal tone and increased sympathetic activity, and the balance of sympathetic and parasympathetic tone regulation was disrupted when schizophrenia patients experienced stress stimulation.

Studies have shown that short-term psychological stress test tends to cause a decrease in HRV (23). Our study suggested that patients had similar responses to metal stress compared with normal controls (25). However, the VLF of normal controls in mental stress represented a highly significant difference compared with that in the resting state. We did not find the same change in schizophrenia patients. The VLF represents the long-term regulation of heart rate, which is related to multiple factors (21). The inhalation and exhalation phases of the respiratory cycle in deep breathing are regarded as changes in the heart period. Experimental results showed that the HF of normal controls significantly changed with breathing frequency compared with that of the resting state. This finding was consistent with the expected normal physiological response to deep breathing and is an indication of a parasympathetic increase, more likely coupled with a decrease in sympathetic activity. However, we did not observe these changes in schizophrenia patients. Therefore, the autonomous system of schizophrenia patients did not function normally under the parasympathetic stimulation. The results demonstrated that the respiration model of schizophrenia patients had been altered and cardiorespiratory coupling decreased. In this study, an additional test of recovery regarded changes in the autonomic system after the period of stress. Notably, the recovery of LF, HF and ApEn was noticeable in normal controls, whereas these features of schizophrenia patients did not show a significant change immediately after deep breathing cessation. Parasympathetic afferent stimulation leads to reflex activation of vagal activity and the inhibition of sympathetic activity (49). The prolonged decrease in ApEn indicated prolonged stress activation. At the same time, the reduced complexity and non-stationarity of heart rate are attributed to autonomic dysregulation of schizophrenia patients. The findings in SD2 of normal controls in the ANS test indicated that SD2, to some extent, positively reflected parasympathetic activity and was strongly influenced by vagal tone. However, the SD2 of schizophrenia patients did not noticeably change in the ANS test. The reduction of long-term variability resulted in reduced sensitivity for the Poincaré indices with respect to the ANS test. According to the results of the between-group study, a highly significant difference was noted, and the SD2 parameter of schizophrenia patients was absolutely abnormal. In total, changes in HRV parameters of normal controls during recovery indicated the balance of sympathetic and parasympathetic tone influences. However, the results of patients demonstrated that the interaction function between two branches of the autonomic system was lopsided, and the tension of the parasympathetic tone was impaired, and the activation of the sympathetic system was inhibited. The schizophrenia patients could not recover normally from the stress as normal controls could.

There are several limitations in this study. First, we did not include schizophrenia patients that were not taking drugs. We have collected the data of medications in schizophrenia patients, such as chlorpromazine equivalent. However, we didn't include it as a covariant in the statistical analysis. Drugs have been suggested to have an effect on the autonomic system, especially the parasympathetic system (50, 51). However, these findings are not replicated and are barely verified in clinical applications. The studies of schizophrenia patients on polypharmacy has proven to be common. Several studies have demonstrated that antipsychotic drugs poorly impact HRV of schizophrenia patients (13). Second, although the computer player guided the operation of subjects in the ANS test, a less cooperative case might affect the results. Moreover, we monitored the operator of all subjects during the ANS test, and all of them seemed to regard the test seriously. Third, we did not assess the relationship between the severity of clinical symptoms and HRV due to the lack of a Positive and Negative Syndrome Scale (PANSS) score. Future studies should systematically explore the association of PANSS scores and HRV parameters, as it may serve as an indicator of the severity of schizophrenia. What's more, future studies should collect neuropsychological tests, such as the level of stress in both groups measured by the questionnaire method (e.g., Perceived Stress Questionnaire), mental health problems in healthy controls measured by Beck Depression Inventory or Beck Depression Inventory, as there may be interesting discoveries.

Our results demonstrated that both the linear and nonlinear features of HRV of schizophrenia patients under long-term stress exhibited a prolonged autonomic activation response after stress source cessation. Measures of the dynamic parameters of HRV were confirmed to be as sensitive to the stress test as linear parameters. This result indicated that not only the time series but also the complexity of HRV and the RR interval variability of schizophrenia patients were decreased compared with those of normal controls. Larger patient samples and tests should be applied to investigate the effect and meaning of dynamic parameters on the described findings in future studies.

## Conclusion

Our results demonstrated that both the linear and nonlinear features of HRV of schizophrenia patients under long-term stress exhibited a prolonged autonomic activation response after stress source cessation. Measures of the dynamic parameters of HRV were confirmed to be as sensitive to the stress test as linear parameters. This result indicated that not only the time series but also the complexity of HRV and the RR interval variability of schizophrenia patients were decreased compared with those of normal controls. Larger patient samples and tests should be applied to investigate the effect and meaning of dynamic parameters on the described findings in future studies.

## Data Availability Statement

The raw data supporting the conclusions of this article will be made available by the authors, without undue reservation.

## Ethics Statement

The studies involving human participants were reviewed and approved by Affiliated Brain Hospital of Guangzhou Medical University. The patients/participants provided their written informed consent to participate in this study.

## Author Contributions

YL conducted the data processing, manuscript preparation, and modification. KW, JZ, and GL developed the study design and participated the manuscript modification. FW, YH, and JC supervised the subject selection and data acquisition. All authors contributed to the article and approved the submitted version.

## Conflict of Interest

The authors declare that the research was conducted in the absence of any commercial or financial relationships that could be construed as a potential conflict of interest.
